# Exploring the impact of diabetes on aging: insights from TERT and COL1A1 methylation

**DOI:** 10.55730/1300-0152.2701

**Published:** 2024-06-26

**Authors:** Jessica Nathania LIAMRI, Farizky Martriano HUMARDANI, Giovani CHANDRA, Lisa Thalia MULYANATA, Tjie KOK, Fenny IRAWATI, Hikmawan Wahyu SULISTOMO, Christoph REICHETZEDER, Sulistyo Emantoko DWI PUTRA

**Affiliations:** 1 Faculty of Biotechnology, University of Surabaya, Surabaya, Indonesia; 2 Department of Biomedical Science, Faculty of Medicine, Universitas Brawijaya, Malang, Indonesia; 3 Faculty of Medicine, University of Surabaya, Surabaya, Indonesia; 4 Bioinformatics Research Center, Indonesia Bioinformatics and Biomolecular, Malang, Indonesia; 5 Institute of Clinical Research and Systems Medicine, Health and Medical University, Potsdam, Germany

**Keywords:** Aging, diabetes, marker, methylation, promoter

## Abstract

**Background/aim:**

Aging, a multifaceted biological process, leads to diminished physical performance, especially in older adults with diabetes, where a mismatch between biological and chronological age is noticeable. Numerous studies have demonstrated that diabetes accelerates aging at the cellular and organ levels. Notable aging markers are telomerase reverse transcriptase (TERT), related to telomere length, and type 1 chain collagen (COL1A1), a key component of skin collagen. Additionally, age-related methylation increases, as revealed through methylation analysis, augmenting aspects of aging. However, the detailed interplay between aging and diabetes, particularly regarding methylation, remains underexplored and warrants further study to elucidate the biological links between the two.

**Materials and methods:**

In this study, we elucidate the modulatory influence of diabetes on the aging process, focusing specifically on the modifications in TERT in the kidney and COL1A1 in the skin using mice of Swiss Webster strain as the diabetes model. Specimens were categorized into three distinct chronological cohorts: chronologically young (16 weeks; n = 5), chronologically old (40 weeks; n = 5), and a periodically assessed group (16 weeks; n = 30), from which five mice were systematically sacrificed on a weekly basis.

**Results:**

Our findings reveal a marked impact of diabetes on the methylation statuses of TERT and COL1A1, characterized by an elevation in methylation levels within the periodic group (1st–6th week) and a simultaneous, progressive attenuation in the expression of TERT and COL1A1 genes.

**Conclusion:**

The observed alterations in the methylation levels of TERT and COL1A1 propound the hypothesis that diabetes potentially expedites the aging process, concomitantly impinging on the production of TERT and COL1A, ostensibly through the mechanism of promoter gene hypermethylation.

## 1. Introduction

Aging is a pervasive biological process that causes a progressive and irreversible decline in physical performance due to damage in response to various stimuli. Different concepts are used to describe age. Chronological age represents the age of an organism solely according to the time of birth. In contrast, the concept of biological age (BA) refers to quantifiable changes occurring on a cellular level that can be assessed by the analysis of various biomarkers (Bahour et al., 2022). Different types of diseases are associated with an acceleration of the aging process, leading to disparities between chronological age and BA. A critical disease in this regard is diabetes mellitus (DM). Previous studies have demonstrated that diabetes, irrespective of the underlying pathophysiology, increases BA, leading to a pronounced discrepancy between BA and chronological age in affected individuals (Bahour et al., 2022).

DM, particularly when disease management is inadequate, has deleterious effects on numerous organ systems. A significant secondary complication arising from diabetes is kidney disease, potentially leading to the necessity for renal replacement therapy (Nieto-Martinez et al., 2021; Hill et al., 2023). As outlined in a recent review by Guo et al., diabetic kidney disease is linked to hastened kidney aging. An important driver of this accelerated kidney aging is cellular senescence, which leads to increased metabolically active cells that cease to divide (Guo et al., 2020).

A crucial mechanism of cellular senescence is telomere shortening, caused by the gradual loss of nucleotides from the protective nucleoprotein structures at the ends of linear chromosomes during cell division. Telomere shortening is a hallmark of aging (Hill et al., 2023). Cells with a high proliferative index, such as stem cells, delay cellular senescence by activating the telomerase enzyme, elongating telomeres via a series of sequenced replications (Hill et al., 2023), whereas somatic cells usually do not exhibit telomerase activity. Diminution in telomere length can be precipitated by a reduction in telomerase reverse transcriptase (TERT) expression, a phenomenon observed in both aging processes and diabetic conditions (Levstek and Trebušak Podkrajšek, 2023).

Several genes encoding proteins implicated in aging have been identified, with two exhibiting significant potential as markers of aging: gene encoding TERT and gene encoding type 1 chain collagen (COL1A1). TERT expression is elevated in cancer but remains low in most tissues (Momeni-Boroujeni et al., 2022). Studies have shown that the adult kidney possesses inherent regenerative abilities that involve the protein component of telomerase. Surprisingly, transient overexpression of TERT can initiate significant podocyte proliferation and renewal (Montandon et al., 2022).

Diabetes manifests itself through damage to both large and small blood vessels, potentially leading to heart attacks and strokes, along with complications in the kidneys, eyes, feet, and nerves. Although the skin is often overlooked as a site of diabetic complications due to its apparent lack of harm, diabetes can prompt premature aging of the skin (Noordam et al., 2013; Giri et al., 2018). This signifies that diabetes acts as a catalyst for aging processes, inducing accelerated aging at multiple biological scales, ranging from cellular to organ levels.

In contrast, the skin, which is easily visible to the naked eye, acts as an important marker of aging. Type I collagen, which forms about 80% of the skin’s total collagen, primarily consists of two α1 (COL1A1) chains and one α2 (COL1A2) chain (Amirrah et al., 2022). It has been observed that the expression of COL1A1 in the skin decreases with aging (Lago and Puzzi, 2019). Studies have shown that collagen production in the skin of rats with diabetes induced by streptozotocin (STZ) drops by 42% (Spanheimer et al., 1988). The acceleration of the aging process in the skin is linked to diabetes, which heightens oxidative stress; external factors like air pollution, inadequate diet, and tobacco use further influence it (Ibuki et al., 2018; Wong and Chew, 2021).

Methylation analysis is a viable method for exploring aging processes, given its significant association with aging mechanisms. More specifically, advancing age is correlated with increased levels of methylation (Zhang et al., 2019). However, existing methodologies primarily focus on whole-genome methylation; the mechanisms of COL1A1 and TERT in diabetes-induced aging are not entirely understood. To address these methodological and knowledge-related gaps, this study aims to assess methylation markers such as COL1A1 and TERT, explore their potential as indicators for diabetes-induced aging, and investigate the underlying mechanisms of aging impacting these markers.

## 2. Materials and methods

### 2.1. Study design and protocol

The Institutional Ethical Committee of the University of Surabaya considered the research design ethically justifiable, evidenced by its acceptance of the research and issuance of a corresponding ethical clearance (No. 226/KE/XII/2021). This study involved a sample of 40 male mice, aligning with the specified criteria detailed in Table 1. The mice experienced a 1-week acclimatization period after being placed into separate enclosures. The environmental lighting was manipulated to simulate a diurnal cycle, alternating between 12 h of light and 12 h of darkness.

**Figure 1:**
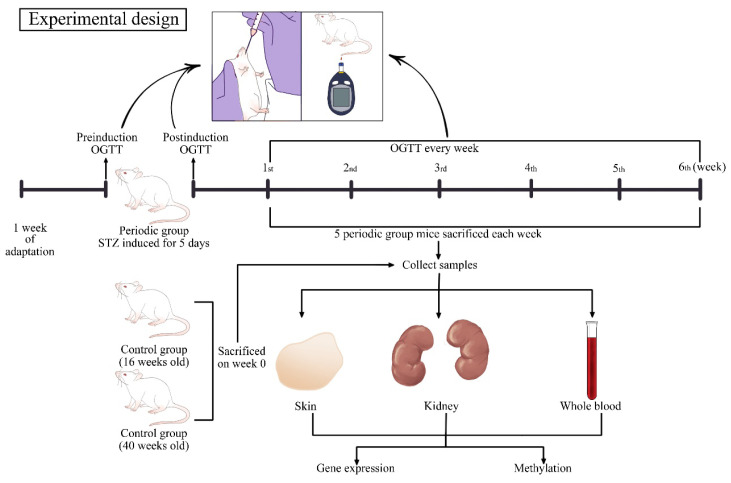
Experimental design. Following a week of acclimatization to the new environment, the mice were divided into three large groups. STZ was administered to the treated mice to induce diabetes. OGTT was performed once a week for weekly observation; 5 mice were sacrificed weekly to collect kidneys, skin, and blood. Methylation analysis was performed on skin, kidney, and blood samples, followed by gene expression analysis on kidney and skin samples.

The duration of the study was 6 weeks, during which the mice were categorized into three distinct groups: five chronologically young mice (16 weeks old), five chronologically old control mice (40 weeks old), and 30 mice, called the periodic group, aged 16 to 22 weeks. The selection of mice aged 16 to 22 weeks was intended to facilitate a comparison between young and old mice in terms of methylation differences. This age range also helped in understanding how progressive diabetes contributes to increased methylation over several weeks. Observations were carried out on the periodic group over 6 weeks. From the 1st to the 6th week, five mice from the periodic group were systematically sacrificed each week via cervical dislocation to procure kidney and skin tissues and whole blood samples from the heart (Figure 1).

### 2.2. Animal diabetes model

Diabetes was induced in the periodic group, while the control group (both young and old mice) did not undergo diabetes induction. Diabetes induction was achieved by administrating low doses of STZ (40 mg/kg per body weight for 5 consecutive days) via intraperitoneal injection using a 1-mL syringe equipped with a 25G 1/2-inch needle for the periodic group. STZ-induced diabetes was chosen for this study due to its well-established methodology and shorter induction time than diet-induced diabetes, which requires 14 weeks to confirm diabetic status, as demonstrated in one of our previous studies (Putra et al., 2023). The following formula was considered for injection volume per individual mouse:


$$SYZ\;volume\frac{mouse\;weight\;(g)}{1000}\times STZ\;dosage(\frac{mg}{kg})$$

The dosage of STZ was precisely determined based on the individual weight of each mouse. Administration involved a partial syringe insertion at a 10-degree angle into the lower quadrant of the abdomen, subsequently adjusted to a 90-degree angle to achieve full insertion. Sodium chloride was administered to the mice as a control substance. An oral glucose tolerance test (OGTT) was given to all groups at both preinduction and postinduction of STZ. For this procedure, the mice were subjected to a 6-h fasting period, followed by oral glucose administration at 2 g/kg per body weight. Subsequent blood glucose levels were quantified from the tail vein at intervals of 0, 15, 30, 60, and 120 min, utilizing a glucose meter (Auto-check, PT Mega Pratama Medicalindo, Indonesia). A 6-h fasting period was selected due to its demonstrated superiority over other durations, as fasting for longer than 6 h has been shown to increase insulin sensitivity (Benedé-Ubieto et al., 2020).

### 2.3. Sample preparation, bisulfite conversion treatment, and primer design

Whole blood was collected via heart puncture into tubes containing EDTA, and kidneys were harvested and stored in 1.8-mL microcentrifuge tubes. RNA was isolated from both kidney and skin tissues, while DNA was extracted from kidney, skin, and whole blood samples. The FavorPrep Tissue Genomic DNA Extraction Mini Kit (Favorgen, Ping Tung, Taiwan) was utilized for DNA extraction, and the FavorPrep Tissue Total RNA Mini Kit (Favorgen, Ping Tung, Taiwan) was employed for RNA extraction, with both RNA and DNA extracted according to the kit’s protocols. Postisolation, the DNA samples were subjected to bisulfite conversion treatment. Methylation-specific PCR **(**MSP) primers were meticulously designed utilizing the primer designing tool Primer-BLAST available from the NCBI.^1^

### 2.4. Methylation-specific PCR

Postbisulfite-treated DNA was amplified utilizing nested PCR. Subsequently, the resulting nested PCR products were further amplified via MSP, employing methylated and unmethylated primers targeting the promoter regions of TERT and COL1A1 (Table 2). Postamplification, the amplicons were segregated through electrophoresis, and their morphologies were assessed using geldoc. The intensity of the resultant bands was quantitatively analyzed with AlphaEaseFC (Alpha Innotech, San Leandro, CA, USA) to determine the methylation percentage.

### 2.5. Gene expression

RNA was transcribed into cDNA utilizing the ExcelRT Reverse Transcription Kit II (SMOBIO Technology, Hsinchu City, Taiwan). Subsequently, the cDNA was amplified employing PCR with primers, as detailed in Table 3. Postelectrophoresis, the amplicon was visualized through GelDoc. The intensity of the bands was analyzed using AlphaEaseFC. Relative gene expression was calculated by normalizing the intensity of the target band to that of the glyceraldehyde 3-phosphate dehydrogenase band, employing the method described by Hazman (2022). This approach uses a cost-effective alternative to RT-qPCR to determine gene expression. The formula used is as follows:


$$Gene\;expression=\frac{\Delta \frac{intensity\;of\;reference\;band}{intensity\;of\;target\;band}target}{\Delta \frac{intensity\;of\;reference\;band}{intensity\;of\;target\;band}control}$$

### 2.6. Pancreas histology

Mice were selected from the chronologically young group and the 6-week observation periodic groups (22 weeks old). The selected mice were euthanized and dissected for further analysis. The harvested pancreases were rinsed with 0.9% NaCl before being immersed in a container with 50 mL of 10% formalin.

### 2.7. Data analysis

The dataset was statistically analyzed using SPSS Statistics 25 (IBM Corp., Armonk, NY, USA). The data from these experiments were collected in triplicate to enhance both robustness and reliability, thereby reducing the impact of any potential anomalies or inconsistencies. The distribution of the data from the OGTT measurements, methylation percentages, and relative expressions was evaluated with the Shapiro–Wilk test before using a One-Way Analysis of Variance to evaluate the data, with further examinations conducted via the Bonferroni post hoc test for multiple comparisons. The Pearson correlation test was employed to ascertain the interrelationships between AUC, methylation percentages, and gene expression levels. Graphs were constructed utilizing GraphPad Prism 9 (GraphPad Software, Boston, MA, USA).

## 3. Results

### 3.1. Glucose profile

**Figure 2:**
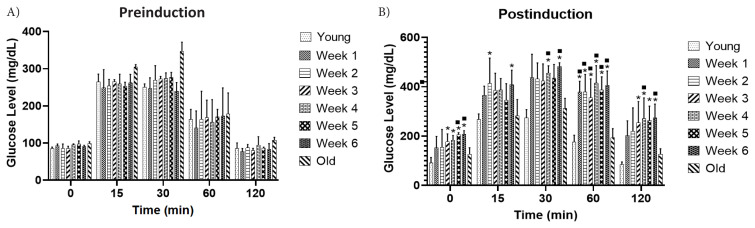
Oral glucose tolerance test between young control mice, old control mice, and treatment group mice before induction (a). Comparisons were made between the OGTT results of the treatment group after induction with the baseline glucose levels of the control group (b); (* = p < 0.05 from the young control group; ▪ = p < 0.05 from the old control group).

Before induction, all mice underwent an OGTT, as shown in Figure 2a, which revealed that none were diabetic; all mice had blood glucose levels within the normal range. Following the induction, another OGTT was administered subsequent to STZ-induction. Mice in the periodic group were confirmed as diabetic (Figure 2b). Diabetes was diagnosed when the mice demonstrated fasting blood glucose levels exceeding 150 mg/dL and glucose levels above 150 mg/dL at 120 min postglucose administration (Logan et al., 2020).

**Figure 3:**
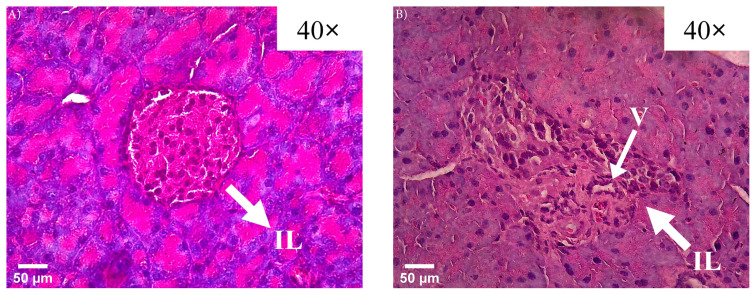
Islets of Langerhans differences in the young control (a) and periodic groups (6th week) (b) at a magnification of 40×. The arrows show islets of Langerhans (IL) and islets of Langerhans of the periodic group suffering from hypertrophy and vacuolization (V). Scale bar = 50 μm.

We conducted a histological analysis of the pancreas using hematoxylin and eosin staining to validate whether the periodic group (at the 6th week) exhibited signs of diabetes compared to the young control group. This analysis enabled an observation of histological alterations in the pancreas to corroborate the presence of diabetes in the treatment group mice. Figure 3a illustrates a healthy pancreas, while Figure 3b displays islets from 6-week-old diabetic mice demonstrating both amorphous enlargement (hypertrophy) and increased cellularity (hyperplasia).

### 3.2. Impact of diabetes on the methylation levels of TERT and COL1A1

**Figure 4:**
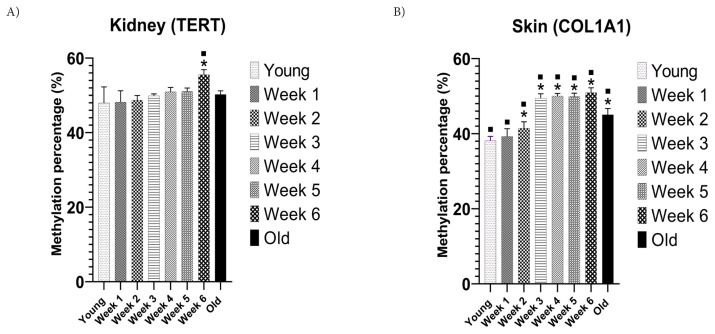
TERT and COL1A1 methylation in kidney (a) and skin (b); (* = p < 0.05 from the young control group; ▪ = p < 0.05 from the old control group).

To assess the impact of diabetes on methylation levels, we contrasted the periodic group (from the 1st to the 6th week) with the chronologically young and old control groups. Our findings indicate that, in the periodic group, the methylation levels of TERT in the kidney and whole blood incrementally increased as diabetes progressed. Specifically, methylation of TERT in the kidney was 5% higher (p < 0.05) in the periodic group (6th week) than in the chronologically old control group and 7% higher than the young control group (Figure 4a). The advancement of diabetes was synchronized with COL1A1 hypermethylation. The methylation percentage of COL1A1 in the skin increased by 6% (p < 0.05) in the periodic group (6th week) relative to the old control group. It was 13% higher than the young control group (Figure 4b). These results imply that diabetes accelerates the biological aging of the kidney and skin.

TERT exhibited notable hypermethylation, being 5% higher in the whole blood of the periodic group at the 6th week than in the whole blood of the old mice control group (p < 0.05; Figure S1a). The methylation percentage of COL1A1 in the whole blood increased by 15% (p < 0.05) in the periodic group in the 6th week compared to the young control group (Figure S1b). The old control group showed hypomethylation compared to the young control and periodic groups (Figure S2a). There is a significant positive correlation (r = 0.561) between the methylation levels of TERT in the kidney and whole blood. Moreover, results produced by COL1A1 did not accurately reflect those of whole blood (Figure S2b); specifically, the old mice control group exhibited hypermethylation in whole blood samples (Figure S1b) and hypomethylation in skin tissue samples (Figure 4b).

### 3.3. Impact of diabetes on TERT and COL1A1 gene expression

**Figure 5:**
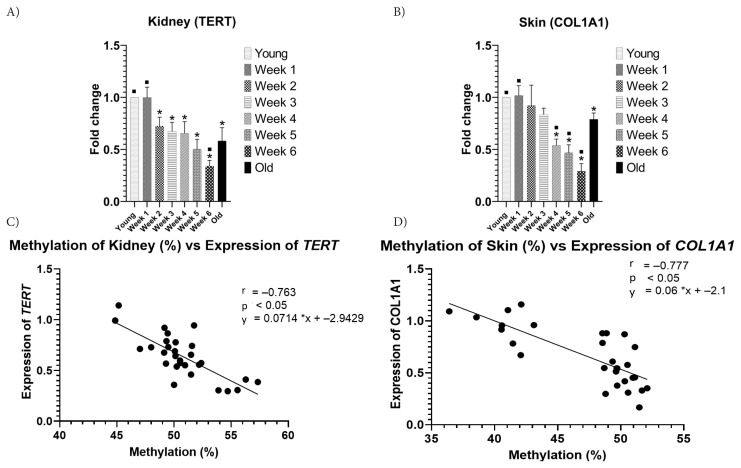
TERT and COL1A1 gene expression. TERT expression (a) and COL1A1 expression (b). Correlations between the methylation percentage and relative expression of TERT (c) and COL1A1 (d); (* = p < 0.05 from the young control group; ▪ = p < 0.05 from old control).

To elucidate the impact of diabetes on gene expression in the kidney and skin, we examined the expressions of TERT and COL1A1. The expression levels of TERT and COL1A1 experienced a gradual decline in the periodic group, exhibiting a significant difference compared to the young and old control groups (p < 0.05) (Figures 5a and 5b). A significant negative correlation was observed between the methylation levels of TERT in the kidney and COL1A1 in the skin and their corresponding gene expressions (Figures 5c and 5d). Similar findings were also reported for whole blood samples for both TERT and COL1A1 (Figures S3a and S3b).

## 4. Discussion

In this study, TERT and COL1A1 were utilized as markers to evaluate the implications of diabetes on aging. The reduction in telomere length, a marker of aging, can be quantified by the diminished expression of TERT, and a decline in COL1A1 expression is correlated with skin aging. It has been previously established that an augmentation in aging correlates with increased methylation (Zhang et al., 2019). Our findings suggest that diabetes triggers enhanced methylation of the TERT and COL1A1 promoters. Examination of the periodic group disclosed a trend of incremental methylation level from the 1st to the 6th week. Concurrent with the methylation levels, the expressions of TERT and COL1A1 also diminished. These results substantiate the proposition that diabetes accelerates the progression of premature aging.

Methylation of the TERT promoter has been extensively studied, especially in various cancers, and is often characterized by hypermethylation (Lee et al., 2021). Our findings indicate that this hypermethylation also occurs in diabetes-induced aging, which might influence telomere dynamics. This phenomenon may result in the shortening of telomeres due to the decreased expression of TERT, likely driven by the hypermethylation of its promoter. It is pertinent to note that chronic inflammation, such as that triggered by *H. pylori*, leads to the production of reactive oxygen species (ROS) (Bussière et al., 2019). Similarly, diabetes enhances the production of ROS, potentially fostering an environment conducive to hypermethylation, which may, in turn, result in diminished gene expression (Volpe et al., 2018).

DNA methylation can modulate gene expression by decreasing DNA accessibility and inhibiting the binding of transcription factors (Kaluscha et al., 2022). As a result, diminished TERT expression can lead to telomere shortening. This telomere shortening can prompt β cells within the islets of Langerhans to attempt self-repair. The incapability of telomeres to mend single-strand DNA breaks, stemming from oxidative or alkylative DNA damage, results in rapid telomere shortening. The underlying reason telomeres fail to repair such single-strand breaks remains elusive (Von Zglinicki, 2002).

The expression of COL1A1 in the skin has been observed to diminish with age (Lago and Puzzi, 2019). Research reveals a 42% reduction in collagen production in the skin of STZ-induced diabetic rats (Spanheimer et al., 1988). Moreover, studies involving mice exhibiting resistance to COL1A1 demonstrated significantly impaired wound healing (Beare et al., 2003). Our data indicate a consistent decline in COL1A1 expression levels in diabetic mice, correlated with elevated glucose levels and the progression of diabetes. This phenomenon may be attributed to hypermethylation occurring in the COL1A1 promoter. A positive correlation was identified between the methylation levels of TERT and COL1A1 in the whole blood and tissue samples, suggesting the potential of using whole blood as an alternative sample source in future studies.

**Figure 6:**
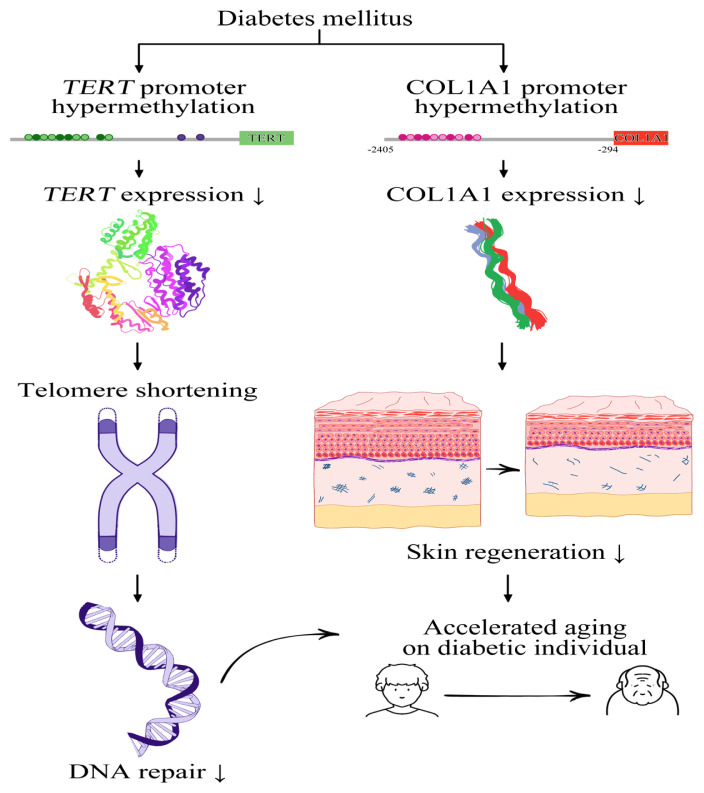
Impact of diabetes on aging processes. Diabetes mellitus is implicated in accelerated aging, primarily through the hypermethylation of telomerase reverse transcriptase (TERT) and collagen type I alpha 1 chain (COL1A1) promoters. This hypermethylation leads to a series of cellular and molecular alterations, including the shortening of telomeres and compromised DNA repair mechanisms, which are integral components of cellular aging. Specifically, the shortened telomeres induce a repair response in β cells located within the islets of Langerhans. Furthermore, this metabolic condition is associated with diminished collagen synthesis, a hallmark of aging, especially in elevated blood glucose levels prevalent in diabetes mellitus. These pathological alterations collectively serve as indicators of premature aging. They can be instrumental in evaluating aging processes and developing potential therapeutic interventions targeting aging-associated pathologies in diabetic populations.

The suggested mechanism proposes that diabetes expedites aging processes through the hypermethylation of TERT and COL1A1 promoters. In individuals with diabetes, this hypermethylation tends to reduce TERT expression, consequently leading to the shortening of telomeres and impairing DNA repair mechanisms, exacerbated by the elevated levels of ROS induced by diabetes. Telomere shortening can prompt β cells within the islets of Langerhans to attempt self-repair. Additionally, a reduced COL1A1 expression, observable in diabetes, correlates with decreased collagen synthesis. This negatively affects skin regeneration and is a precursor of premature skin aging, with a notable correlation to blood glucose levels (Figure 6).

## 5. Conclusion

Diabetes has been shown to increase the methylation of TERT in the kidney and COL1A1 in the skin, leading to reduced expression of these genes and expedited aging. The results derived from tissue methylation are promising and can lead to further research, potentially contributing to the development of noninvasive tools for measuring BA based on tissue-specific markers.

## Supplementary Data

Figure S1 TERT and COL1A1 methylation in blood fo*r* TERT (a) and COL1A1 (b); (* = p < 0.05 from the young control group; ▪ = p < 0.05 from the old control group).

Figure S2 Correlation between methylation of kidney and blood of TERT (a) and methylation of skin and whole blood of COL1A1 (b).

Figure S3 Correlation between methylation and expression of TERT (a) and methylation and expression of COL1A1 (b) in whole blood samples.

## Figures and Tables

**Table 1:** Characteristics of the mice model.

Characteristics	Young control mice	Old control mice	Periodic mice
Number of samples	5	5	30
Strain	Swiss Webster	Swiss Webster	Swiss Webster
Age (weeks)	±16	±40	±16
Initial fasting blood glucose level	84.7 ± 4.2	99 ± 4.2	91.2 ± 11.12
Initial 2-h post-OGTT	85.3 ± 14.8	107.5 ± 7.8	84.6 ± 12.4
Initial weight (g)	24.06 ± 5.9	33.15 ± 4.4	28.13 ± 4.1

**Table 2:** Primer COL1A1 and TERT for MSP.

	Primer	Sequence	Primer Length (bp)	Tm (°C)	Amplicon (bp)
**COL1A1**	MSP *Forward Methylated*	5′ – GATGGTATAAAAGGGGTTTAGGTTAGTC – 3′	28	59.54	150
	MSP *Forward Unmethylated*	5′ – GATGGTATAAAAGGGGTTTAGGTTAGTT – 3′	28		
	MSP *Reverse*	5′ – ATCTAAACCCTAAACATATAAACTCTTTAC – 3′	30	56.43	
	*Nested Forward*	5′ – GGGTTAGGTAGTTTTGATTGGTTGG – 3′	25	60.05	389
	*Nested Reverse*	5′ – AATCTTATCTACTAAAACCCCTCTATAC – 3′	28	56.20	
**TERT**	MSP *Forward Methylated*	5′ – GTAGAGGGAAATTTTGTATGAGTGC – 3′	25	58.21	110
	MSP *Forward Unmethylated*	5′ – GTAGAGGGAAATTTTGTATGAGTGT – 3′	25		
	MSP *Reverse*	5′ – ATCACAATACTAATACATAAAAACCA – 3′	26	53.33	
	*Nested Forward*	5′ – AGGATAGGTTTTTTTGTTTGTTTAA – 3′	25	54.29	342
	*Nested Reverse*	5′ – AAACAACCAAAACCCAAACCACTAA – 3′	25	60.22	

**Table 3:** Primer for gene expression.

	Primer	Sequence	Primer Length (bp)	Tm (°C)	Amplicon (bp)
**COL1A1**	*Forward* qPCR	5′ – GCCTTGGAGGAGTTCTTTATAG –3′	22	55.96	209
	*Reverse* qPCR	5′ – TAACCAGACAATCCCAAACTAC – 3′	22	55.73	
**TERT**	*Forward* qPCR	5′ – GGAAAGATAAATGGGAAGGACCG – 3′	23	59.12	155
	*Reverse* qPCR	5′ – CTGTTCTTCTGGAATGTGCTCTC – 3′	23	59.32	
**GAPDH**	*Forward* qPCR	5′ – GTCAAGGCCGAGAATGGGAA – 3′	20	60.04	79
	*Reverse* qPCR	5′ – TGATGTTAGTGGGGTCTCGC – 3′	20	59.46	

## Footnotes

1 NCBI (2012). Primer-BLAST [online]. Website: https://www.ncbi.nlm.nih.gov/tools/primer-blast/ [accessed 13 January 2022].
