# Supplementary figures and images for: Exploring the impact of diabetes on aging: insights from TERT and COL1A1 methylation
Source: Turk J Biol. 2024 Jun 26;48(4):257–66. doi: 10.55730/1300-0152.2701 (PMC11407328; doi:10.55730/1300-0152.2701)

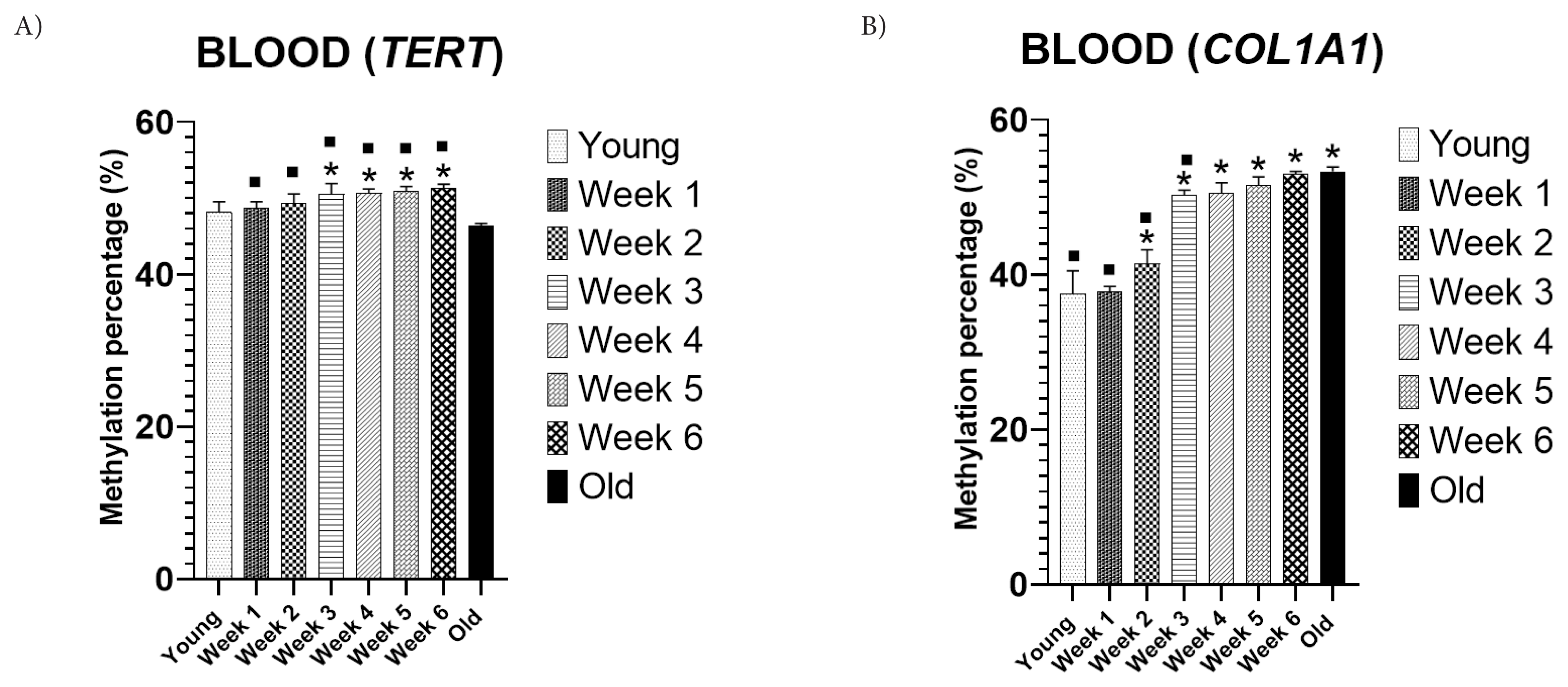

Supplement: Figure S1 — TERT and COL1A1 methylation in blood for TERT (a) and COL1A1 (b); (* = p < 0.05 from the young control group; ▪ = p < 0.05 from the old control group). [file tjb-48-04-257s1.tif]

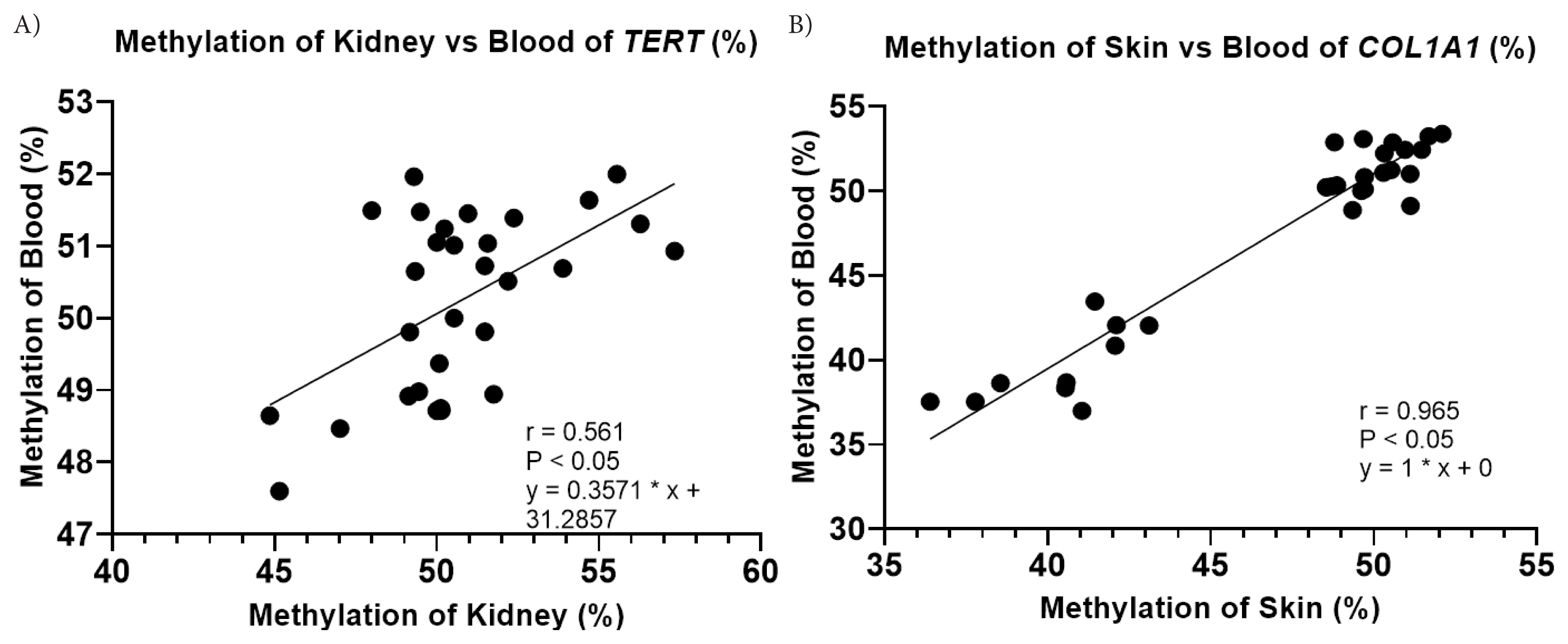

Supplement: Figure S2 — Correlation between methylation of kidney and blood of TERT (a) and methylation of skin and whole blood of COL1A1 (b). [file tjb-48-04-257s2.tif]

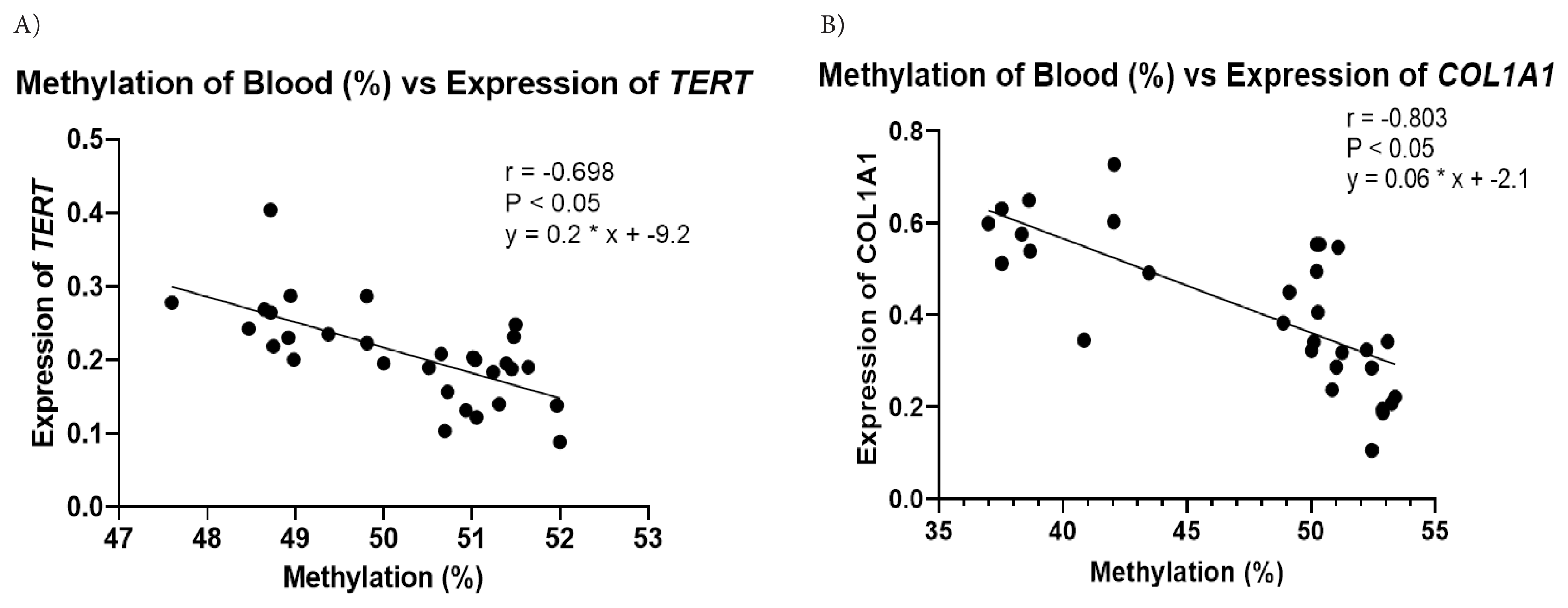

Supplement: Figure S3 — Correlation between methylation and expression of TERT (a) and methylation and expression of COL1A1 (b) in whole blood samples. [file tjb-48-04-257s3.tif]
